# Risk factors affecting cataract surgery outcome: The Malaysian cataract surgery registry

**DOI:** 10.1371/journal.pone.0274939

**Published:** 2022-09-21

**Authors:** Geng-Yi Yong, Jelinar Mohamed-Noor, Mohamad Aziz Salowi, Tassha Hilda Adnan, Mimiwati Zahari

**Affiliations:** 1 Department of Ophthalmology, Kuala Lumpur Hospital, Kuala Lumpur, Malaysia; 2 Department of Ophthalmology, University of Malaya, Kuala Lumpur, Malaysia; 3 Department of Ophthalmology, Hospital Selayang, Batu Caves, Malaysia; 4 Faculty of Medicine, Universiti Sultan Zainal Abidin, Kuala Terengganu, Malaysia; 5 Biostatistics Unit, National Clinical Research Centre, Kuala Lumpur, Malaysia; University of Warmia, POLAND

## Abstract

This is a 5 years multicentre database study that recruited subjects from the Malaysian Ministry of Health Cataract Surgery Registry (MOH CSR), aimed to determine risk factors that affect cataract surgery visual outcome and evaluates post-cataract surgery vision. All age-related cataract surgeries with primary intraocular lens (IOL) implantation were included. Cases with secondary cataract, previous ocular surgeries and incomplete data were excluded. A total of 131425 cases were included in the study. Amongst all types of cataract surgery, 92.9% attained post-operative best-corrected visual acuity better than 6/18 and the outcome improved to 97.1% when ocular comorbidities were excluded. Factors with Odds Ratio (OR) >1.5 associated with an impaired visual outcome included: elderly patients of 80 years old and above; systemic disease such as renal failure; ocular co-morbidities; pre-operative vision worse than 6/60; general anaesthesia, retrobulbar anaesthesia or subconjunctival anaesthesia; extracapsular cataract extraction (ECCE), intracapsular cataract extraction (ICCE), anterior chamber intraocular lens (ACIOL) implantation or combined cataract surgery; the presence of intra- and post-operative complications. In conclusion, a good visual outcome was achieved after cataract surgery in most cases. This large multicentre study provides information about risk factors for poor visual outcome post-cataract surgery and may serve as a basis for evidence-based guidelines.

## Introduction

Cataract is the leading cause of preventable blindness and it comprises 51% blindness worldwide [1]. According to the Malaysia National Eye Survey II in 2014, most of the blindness and visual impairment was contributed by cataract, comprising 58% and 68% respectively [2].

Cataract surgery is the most performed ophthalmic procedure to reduce the burden caused by blindness. Costs from cataract surgery have been estimated at United States dollars (USD) 10.7 billion per year [3]. It is a safe and effective surgical intervention. However, complications arise from the surgery can have a significant impact on visual outcome and additional surgical cost of managing complications range from USD 400 to USD 6000 for an episode of care [4].

The success of cataract surgery depends on the surgical outcome, namely the improvement in visual acuity (VA). Identifying risk factors that determine cataract surgery visual outcome is crucial to improve the standard of cataract surgery by enabling a surgeon to take extra precautions preoperatively or intraoperatively to reduce surgical complications. This will ultimately benefit individuals suffering from cataract globally by reducing the economic burden, improving postoperative visual outcomes, and patients’ satisfaction.

The commonly reported risks for poor visual outcome after cataract surgery were related to age, presence of ocular comorbidities, biometry techniques, the complexity of cataract surgery, and the presence of intraoperative complications [5–9]. The International Cataract Surgery Outcome Study which included the United States of America, Canada, Denmark, and Spain reported a postoperative VA of 6/18 or better in 86% of operated eyes [10]. Moreover, the United Kingdom National Ophthalmology Database Audit and the European Registry of Quality Outcomes for Cataract and Refractive Surgery reported a postoperative VA of 6/12 or better in 91% and 94.3% of operated eyes, respectively [11, 12]. However, when only cases without ocular comorbidities were taken into consideration, 92% - 95% cataract patients were able to achieve a postoperative VA of 6/12 or better [11, 12]. On the other hand, Asia countries like China and Singapore reported postoperative VA of 6/18 or better in 89% of operated eyes [13, 14].

In this large-scale study, we investigated risk factors that affect cataract surgery visual outcome in Malaysia for over 5 years. The study also evaluates post-cataract surgery best-corrected visual acuity (BCVA) among 3 types of cataract surgeries [phacoemulsification (phaco), extracapsular cataract extraction (ECCE) and intracapsular cataract extraction (ICCE)] including patients, with or without ocular comorbidity.

## Materials and methods

This is a multicentre database study. Data were extracted from the Malaysian Ministry of Health Cataract Surgery Registry (MOH CSR) between 1^st^ January 2013 and 31^st^ December 2017 who underwent cataract surgeries in all MOH hospitals (total of 36 hospitals) with ophthalmology services. Ethical approval was acquired from the Malaysia medical research and ethics committee of the MOH and was registered in National Medical Research Register (NMRR-17-2847-38466). We are reporting a retrospective study of medical records or archived samples and all data were fully anonymized before we accessed them. Therefore, Malaysia medical research ethics committee waived the requirement for informed consent. The study was compliant with the guidelines of the Declaration of Helsinki.

All age-related cataract surgeries with primary intraocular lens (IOL) implantation were included. Cases with secondary cataract, previous ocular surgeries, and incomplete data were excluded. The data collected included the patient’s demographic data, systemic and ocular comorbidities, pre-operative visual acuity, and biometry measurement. Intra-operative data was documented which consisted of the date of cataract surgery, surgeon status, types of anaesthesia, types of cataract surgery, location of cornea wound placement, choices of IOL, and intraoperative complications if any. Post-operative VA between 8–12 weeks, data on complications, and their associated factors were recorded. The collected data was divided into two groups namely the good vision group (VA > 6/18) and the impaired vision group (VA ≤ 6/18).

Statistical analysis was performed using Statistical Package for Social Science (SPSS), version 21.0 (SPSS, Inc., Chicago, III., USA) for Windows. The simple logistic regression model was used to estimate the OR for demographic data, pre-operative, intra-operative, and post-operative data. ORs indicating the effect of the risk factors on the poor visual outcome after cataract surgery was calculated and reported with 95% confidence interval (CI). The multiple logistic regression including all the risk factors. Adjusted (Adj.) OR and it’s 95% CI were used to estimate a risk score for combinations of risk factors. The results of the multivariable logistic regression were for factors significant at the 5% level, adjusted for baseline VA. Large sample size and a relatively common outcome resulted in the statistical power of this model were high. Therefore, caution was exercised when interpreting small effects with limited statistical significance such as 0.67<OR<1.5 and/or 0.05<p<0.01 as these may be clinically unimportant.

## Results

A total of 222588 cases were extracted from the database. The total number of cases that fulfilled the inclusion criteria for the study period of 5 years was 131425 cases. Among these, 92.9% of cases had BCVA better than 6/18 (n = 122081), while 7.1% of cases had BCVA 6/18 or worse (n = 9344). When ocular comorbidities were excluded, 97.1% of cases attained post-operative BCVA better than 6/18 (Table 1).

**Table 1:** Post-operative BCVA of all cases and cases without ocular comorbidity.

Outcomes	All patients (n = 131,425)	Patients excluding ocular co-morbidities (n = 82,443)
	n (%)	n (%)
**(A) Postoperative BCVA**		
Good (>6/18)	122,081 (92.9)	80,081 (97.1)
Borderline (6/18–6/60)	6,720 (5.1)	1,929 (2.3)
Poor (<6/60)	2,624 (2.0)	433 (0.5)
>6/12	116,347 (88.5)	78,034 (94.7)
6/6 or better	54,837 (41.7)	39,179 (47.5)

BCVA = best-corrected visual acuity; based on refracted VA of an operated eye.

The mean age at the time of cataract surgery for post-operative BCVA better than the 6/18 group was 67.1 years and the BCVA 6/18 or worse group was 68.3 years (*p*<0.001). The age groups showed that ≥80 years old categories had a higher risk of post-operative BCVA 6/18 or worse compared with subjects aged 59 years and below (OR = 1.87, *p*<0.001) (Table 2).

**Table 2:** Crude and adjusted ORs for post-operative vision worse than 6/18 among all cases from the logistic regression model, 2013–2017 (n = 131425).

Risk factor	Post-op BCVA group		Simple logistic regression		Multivariate logistic regression (n = 114,271)	
	VA ≤ 6/18 (n = 9,344)	VA > 6/18 (n = 122,081)				
	n (%)	n (%)	Crude OR (95% CI)	*p*-value	Adj. OR (95% CI)	*p*-value ^b^
**Age at surgery**:				<0.001		<0.001
50–59 years	1,615 (17.3)	19,875 (16.3)	1.00	-	1.00	-
60–69 years	3,240 (34.7)	51,712 (42.4)	0.77 (0.72, 0.82)	<0.001	0.99 (0.92, 1.07)	0.767
70–79 years	3,271 (35.0)	42,496 (34.8)	0.95 (0.89, 1.01)	0.086	1.42 (1.32, 1.54)	<0.001
≥80 years	1,218 (13.0)	7,998 (6.6)	1.87 (1.73, 2.03)	<0.001	2.71 (2.45, 2.98)	<0.001
**Gender**:						
Male	4,237 (45.3)	56,766 (46.5)	1.00	0.031	1.00	0.001
Female	5,107 (54.7)	65,315 (53.5)	1.05 (1.01, 1.09)		1.09 (1.04, 1.14)	
**Ethnicity**:				<0.001		<0.001
Malay	4,174 (44.7)	52,114 (42.7)	1.00	-	1.00	-
Chinese	3,132 (33.5)	40,615 (33.3)	0.96 (0.92, 1.01)	0.123	1.04 (0.98, 1.10)	0.151
Indian	953 (10.2)	13,939 (11.4)	0.85 (0.79, 0.92)	<0.001	0.86 (0.79, 0.94)	0.001
Others	581 (6.2)	8,170 (6.7)	0.89 (0.81, 0.97)	0.010	1.29 (1.16, 1.42)	<0.001
**Systemic co-morbidity**^a^:						
Diabetes mellitus	5,138 (55.0)	54,621 (44.7)	1.53 (1.47, 1.60)	<0.001	1.26 (1.19, 1.34)	<0.001
Hypertension	6,339 (67.8)	80,942 (66.3)	1.09 (1.04, 1.14)	<0.001	0.85 (0.80, 0.90)	<0.001
Ischaemic heart disease	848 (9.1)	10,541 (8.6)	1.06 (0.99, 1.14)	0.119	1.00 (0.91, 1.09)	0.931
Renal failure	656 (7.0)	3,216 (2.6)	2.80 (2.57, 3.06)	<0.001	1.56 (1.40, 1.75)	<0.001
Cerebro-vascular accident	235 (2.5)	1,840 (1.5)	1.69 (1.47, 1.94)	<0.001	1.38 (1.18, 1.63)	<0.001
**Ocular co-morbidity**^a^:						
Pterygium involving cornea	145 (1.6)	1,633 (1.3)	1.16 (0.98, 1.38)	0.084	1.15 (0.95, 1.40)	0.148
Corneal opacity	254 (2.7)	1,100 (0.9)	3.07 (2.68, 3.53)	<0.001	3.63 (3.11, 4.23)	<0.001
Glaucoma	1,119 (12.0)	7,578 (6.2)	2.06 (1.92, 2.20)	<0.001	2.59 (2.40, 2.80)	<0.001
Chronic uveitis	50 (0.5)	191 (0.2)	3.43 (2.51, 4.69)	<0.001	3.71 (2.58, 5.35)	<0.001
Pseudoexfoliation	148 (1.6)	1,243 (1.0)	1.56 (1.32, 1.86)	<0.001	1.23 (1.01, 1.49)	0.037
Phacomorphic	77 (0.8)	224 (0.2)	4.52 (3.49, 5.86)	<0.001	2.77 (2.07, 3.72)	<0.001
Non-proliferative diabetic retinopathy	995 (10.6)	7,800 (6.4)	1.75 (1.63, 1.87)	<0.001	2.11 (1.94, 2.29)	<0.001
Proliferative diabetic retinopathy	1,211 (13.0)	1,916 (1.6)	9.34 (8.66, 10.07)	<0.001	9.42 (8.59, 10.33)	<0.001
Maculopathy	601 (6.4)	1,215 (1.0)	6.84 (6.19, 7.56)	<0.001	4.26 (3.77, 4.82)	<0.001
Vitreous haemorrhage	235 (2.5)	224 (0.2)	14.03 (11.67, 16.87)	<0.001	2.50 (1.95, 3.19)	<0.001
ARMD	417 (4.5)	2,553 (2.1)	2.19 (1.97, 2.43)	<0.001	3.20 (2.84, 3.59)	<0.001
Other macular disease (includes hole or scar)	611 (6.5)	745 (0.6)	11.39 (10.22, 12.71)	<0.001	12.88 (11.37, 14.59)	<0.001
Optic nerve disease	133 (1.4)	569 (0.5)	3.08 (2.55, 3.73)	<0.001	2.68 (2.17, 3.31)	<0.001
Retinal detachment	266 (2.8)	228 (0.2)	15.66 (13.1, 18.71)	<0.001	10.17 (8.11, 12.76)	<0.001
Cannot be assessed (due to dense cataract)	1,383 (14.8)	13,782 (11.3)	1.37 (1.29, 1.45)	<0.001	1.55 (1.44, 1.66)	<0.001
Amblyopia	69 (0.7)	196 (0.2)	4.63 (3.51, 6.09)	<0.001	6.79 (5.04, 9.15)	<0.001
**Biometry techniques**:				0.002		0.005
Applanation	1,527 (16.3)	18,429 (15.1)	1.14 (1.06, 1.22)	0.001	1.13 (1.04, 1.23)	0.006
Immersion	6,166 (66.0)	81,286 (66.6)	1.04 (0.98, 1.10)	0.186	1.12 (1.04, 1.19)	0.002
Interferometry laser	1,503 (16.1)	20,610 (16.9)	1.00	-	1.00	-
**Pre-operative corrected distance visual acuity**:				<0.001		0.005
Good (≥6/18)	771 (8.3)	37,776 (30.9)	1.00	-	1.00	-
Borderline (<6/18–6/60)	2,142 (22.9)	28,985 (23.7)	3.62 (3.33, 3.94)	<0.001	3.55 (3.23,3.90)	0.298
Poor (<6/60)	3,122 (33.4)	22,923 (18.8)	6.67 (6.16, 7.23)	<0.001	6.63 (6.02, 7.30)	<0.001
**Surgeon status**:				0.001		<0.001
Specialist	8,136 (87.1)	107,225 (87.8)	1.00	-	1.00	-
Gazetting specialist	511 (5.5)	6,898 (5.7)	0.98 (0.89, 1.07)	0.612	1.07 (0.97, 1.19)	0.190
Medical officer	690 (7.4)	7,774 (6.4)	1.17 (1.08, 1.27)	<0.001	0.83 (0.75, 0.91)	<0.001
**Types of anaesthesia**:						
General anaesthesia	588 (6.3)	4,062 (3.3)	1.95 (1.78, 2.13)	<0.001	1.78 (1.57, 2.03)	<0.001
Local anaesthesia	8,714 (93.3)	117,340 (96.1)	1.00	-	1.00	-
**Local anaesthesia**^a^:						
Retrobulbar	340 (3.6)	429 (0.4)	10.7 (9.26, 12.35)	<0.001	2.40 (1.84, 3.12)	<0.001
Peribulbar	52 (0.6)	310 (0.3)	2.20 (1.64, 2.95)	<0.001	1.33 (0.92, 1.93)	0.135
Subtenon	2,454 (26.3)	22,894 (18.8)	1.54 (1.47, 1.62)	<0.001	1.28 (1.18, 1.39)	<0.001
Subconjunctival	335 (3.6)	3,383 (2.8)	1.30 (1.16, 1.46)	<0.001	1.50 (1.31, 1.71)	<0.001
Topical	5,752 (61.6)	85,802 (70.3)	0.67 (0.64, 0.70)	<0.001	1.21 (1.12, 1.30)	<0.001
Intracameral	1,623 (17.4)	27,598 (22.6)	0.72 (0.68, 0.76)	<0.001	0.89 (0.83, 0.95)	<0.001
**Types of surgery**:				<0.001		<0.001
Phaco	7,378 (79.0)	112,623 (92.3)	1.00	-	1.00	-
ECCE	1,821 (19.5)	9,099 (7.5)	3.05 (2.89, 3.23)	<0.001	2.87 (2.62, 3.14)	<0.001
ICCE	97 (1.0)	174 (0.1)	8.51 (6.63, 10.92)	<0.001	2.88 (2.11, 3.92)	<0.001
**Types of IOL**:				<0.001		<0.001
Posterior chamber	8,788 (94.0)	120,735 (98.9)	1.00	-	1.00	-
Anterior chamber	521 (5.6)	1,145 (0.9)	6.25 (5.62, 6.95)	<0.001	3.24 (2.82, 3.73)	<0.001
Scleral fixated PCIOL	7 (0.1)	42 (0.0)	2.29 (1.03, 5.1)	0.043	1.42 (0.58, 3.49)	0.445
**Surgical wound placement during phacoemulsification**:						
Superior	8,132 (87.0)	101,566 (83.2)	1.36 (1.27, 1.45)	<0.001	1.16 (1.08, 1.25)	<0.001
Temporal	1,138 (12.2)	19,274 (15.8)	1.00	-	1.00	-
**Types of combined surgery**^a^:						
Pterygium excision	39 (0.4)	253 (0.2)	2.03 (1.45, 2.84)	<0.001	1.78 (1.23, 2.58)	0.002
Filtering surgery	38 (0.4)	125 (0.1)	0.25 (0.17, 0.36)	<0.001	2.01 (1.35, 3.00)	0.001
Vitreoretinal	466 (5.0)	346 (0.3)	18.57 (16.13, 21.38)	<0.001	2.88 (2.25, 3.69)	<0.001
**Complications**:				<0.001		<0.001
Intra-operative	1,016 (10.9)	4,219 (3.5)	3.41 (3.17, 3.66)	0.001	2.10 (1.89, 2.35)	<0.001
Post-operative	7,364 (78.8)	4,212 (3.5)	104.08 (98.18, 110.33)	<0.001	61.79 (56.93, 67.06)	<0.001

Post-op BCVA, post-operative best-corrected visual acuity; OR, Odds ratio; Adj. OR, Adjusted odds ratio; CI, Confidence interval; ECCE, extracapsular cataract extraction; ICCE, intracapsular cataract extraction.

^a^ One patient may have multiple parameters; the percentage reported is based on total patients in each BCVA group. The remaining unreported frequency and its percentages are the missing value, and the reference group is “No”.

^b^ All variables were included in the multiple logistic regression.

Diabetes mellitus comprised 45.5% of study subjects’ systemic comorbidity. Consequently, diabetic retinopathy was the most common ocular comorbidity which presented in 9.1% of study subjects. The presence of corneal opacity (OR = 3.07, *p*<0.001), glaucoma (OR = 2.06, *p*<0.001), chronic uveitis (OR = 3.43, *p*<0.001), phacomorphic (OR = 4.52, *p*<0.001), proliferative diabetic retinopathy (OR = 9.34, *p*<0.001), maculopathy (OR = 6.84, *p*<0.001), vitreous haemorrhage (OR = 14.03, *p*<0.001), age-related macular degeneration (OR = 2.19, *p*<0.001), other macula disease (OR = 11.39, *p*<0.001), optic nerve disease (OR = 3.08, *p*<0.001), retinal detachment (OR = 15.66, *p*<0.001) and amblyopia(OR = 4.63, *p*<0.001) were associated with the risk of post-operative BCVA 6/18 or worse.

Biometry techniques such as interferometry laser and immersion were positively associated with post-operative BCVA better than 6/18. In comparison, there was a poorer post-operative visual outcome (OR = 1.14, *p* = 0.001) with the use of the applanation technique.

Phacoemulsification was the most common cataract procedure (91.3%). 93.9% of all phacoemulsification cases and 97.6% after ocular comorbidity was omitted, achieved post-operative vision better than 6/18. Subjects who underwent ECCE (OR = 3.05, *p*<0.001) and ICCE (OR = 8.51, *p*<0.001) had a higher risk of developing post-operative BCVA 6/18 or worse compared with those who had phacoemulsification cataract surgery.

Subjects with combined vitreoretinal and cataract surgery (OR = 18.57, *p*<0.001), general anaesthesia (OR = 1.95, *p*<0.001) and anterior chamber IOL implantation (OR = 6.25, *p*<0.001) showed poorer post-operative visual outcome. Presence of intra-operative complications (OR = 3.41, *p* = 0.001) and post-operative complications (OR = 104.08, *p*<0.001) were also identified as factors associated with post-operative vision 6/18 or worse. Posterior capsular rupture and high astigmatisms are the commonest intra- and post-operative complications that contributed to post-operative BCVA 6/18 or worse.

## Discussion

Malaysia comprises 13 states and 3 federal territories. The estimated population of Malaysia is 32.4million with 6.3 million (19.4%) aged 50 years and above [15]. Healthcare in Malaysia is provided by two systems which comprise the public and private sectors. The public sector under the Malaysian Ministry of Health contributes 75% of hospital beds and is the largest healthcare provider in Malaysia [16].

MOH CSR, which is part of the National Eye Database, is a password-protected, electronic database of cataract surgeries performed at all public and selected private healthcare facilities in Malaysia [17]. This is a large nationwide population-based study that may represent the Malaysian population with 3 major ethnicities. This study aims to identify various risk factors that affect cataract surgery outcome. Data from the MOH CSR between 2013 and 2016 were analysed.

Based on our 5-year demographic data, study subjects were Malay (43.0%), Chinese (33.5%) and Indian (12.5%) ethnicity. The population distribution was representative of the population ratio in Malaysia. The mean population age when cataract surgery was performed was 68 years. This is consistent with previous local data derived from CSR (between the year 2002 and 2011) which reported the mean age for cataract surgery at 64.5 years [18]. Patients aged 70 years and above were identified as a risk factor for the poor visual outcome of 6/18 and worse. Wong *et al*. reported a similar finding among elderly patients with multiple systemic illnesses and ocular comorbidities [19]. They also had a higher risk of intra- and post-operative complications such as posterior capsule rupture, postoperative infection, raised intraocular pressure and corneal oedema due to reduced endothelial cell count which resulted in the poor visual outcome [19].

The Malaysia National Health and Morbidity Survey 2011 and 2015 reported the prevalence of diabetes mellitus, hypertension, renal failure, and cerebrovascular accident (CVA) in Malaysia was 17.5%, 35.3%, 9.07% and 0.7% respectively [20, 21]. In our study, we found that systemic and ocular comorbidities were associated with post-operative BCVA 6/18 or worse. The identified risk factors for poor post-operative visual outcome were diabetes mellitus, hypertension, renal failure, and CVA. These conditions have great implication on microvasculature changes in the eye and potentially leads to devastating consequences after cataract surgery. Microvasculature changes in diabetic, hypertensive and renal failure patients increase the risk of post-operative BCVA 6/18 or worse. The potential sight-threatening causes among diabetic patients were macula oedema, proliferative diabetic retinopathy, posterior capsular opacity, corneal decompensation and higher incidence of postoperative endophthalmitis [22–24]. Hence, preoperative assessment is essential for patients with diabetes mellitus who are planned for cataract surgery. These groups of patients should be counselled such that they do not have unrealistic expectations of post-operative vision following their surgery. In addition, optimisation of the systemic condition is crucial for a better post-operative visual outcome as studies have shown that the significant systemic risk factors for the development of sight-threatening conditions are chronic kidney disease, CVA and hypertension [25–27].

Coexisting eye disease is also an important risk factor that contributes to poor post-operative visual outcome. Our study showed ocular comorbidities such as diabetic retinopathy (23.6%), dense cataract obscuring fundal view (14.8%), glaucoma (12.0%) and macular diseases (11.0%) are the major risk factors that are associated with post-operative BCVA 6/18 or worse.

We also found patients with poor visualization of the fundus due to dense cataract had a higher risk of post-operative BCVA 6/18 or worse (adj. OR = 1.54, *p*<0.001). This could be attributed to undetected or pre-existing sight-threatening ocular diseases, such as diabetic retinopathy or macula pathology. Therefore, earlier cataract surgery should be recommended.

Biometry is vital for accurate IOL calculation. Our study showed that applanation ultrasound biometry was a possible risk factor for post-operative BCVA 6/18 or worse (adj. OR = 1.12, *p* = 0.015). Applanation ultrasound biometry may cause inadvertent compression of the eye by the ultrasound transducer resulting in inaccurate axial length measurements [28–31]. In comparison, optical biometry utilises partial coherence interferometry for ocular imaging and measurements of anterior chamber depth, axial length and intraocular lens power [32, 33]. In addition, this method has less axial length measurement variation due to its non-contact method. Hence, it is better than ultrasound biometry, especially applanation biometry [34]. Immersion biometry, however, is still valid as the ultrasonic waves can penetrate dense central cataracts and other media opacities [35]. In our country, immersion biometry is more widely available. It is as accurate as optical biometry and more economical.

In Malaysia, cataract surgery is done by ophthalmologists, gazetting specialists, and medical officers. Our study showed cases done by medical officers were not associated with post-cataract surgery BCVA 6/18 or worse (adj. OR = 0.83, *p*<0.001). This is because surgery done by less experienced medical officers may have a higher risk for surgical complications. Therefore, straightforward cases were allocated to medical officers. Appropriate case selection with supervision by an ophthalmologist is important for the training of medical officers while not compromising patients’ visual outcome.

The visual outcome of surgery depends on various factors like types of cataract surgery, surgical wound type and placement, types of intraocular lens and types of anaesthesia. Comparison between the two groups showed that phacoemulsification and posterior intraocular lens implantation with temporal, clear cornea incision conducted under local anaesthesia achieved post-operative BCVA better than 6/18.

Studies have shown ECCE and ICCE are associated with higher post-operative astigmatism due to tight suture, more discomfort, more surgical complications and has slower visual recovery. A randomized control trial conducted in the United Kingdom found that phacoemulsification was clinically superior and more effective than ECCE [36]. Phacoemulsification had lesser surgical complications and capsule opacity within 1 year after surgery. The majority achieved an unaided VA of 6/9 or better [36]. Nikose et al. reported that surgically induced astigmatism in the temporal corneal incision group is less than in the superior group and produced a better visual outcome, good optical quality, and greater patient satisfaction [37].

There are three categories of anaesthesia: general, regional (retrobulbar, peribulbar, and sub-Tenon’s), and topical (with or without intraocular anaesthetics) used in cataract surgery. However general anaesthesia is rarely undertaken except in special cases. Eichel et al. reported 10% of cataract surgeries were done under general anaesthesia [38], as compared to 3.6% in our study. We also found that cases were done under general and regional anaesthesia (retrobulbar, subtenon, and subconjunctival) had the poorer post-operative visual outcome. A survey by Leaming among members of the American Society of Cataract and Refractive Surgery (ASCRS) found that the use of general anaesthesia or retrobulbar block has largely been replaced with other types of local anaesthesia, which comprise of peribulbar, sub-Tenon’s and topical anaesthesia. These modalities are equally effective and safer in terms of reducing devastating risks, such as retrobulbar haemorrhage and globe perforation [39]. However, in our study we found that a proportion of cases who had subtenon and subconjunctival anaesthesia were associated with post-operative BCVA 6/18 or worse. This may be because these modalities were used in complicated or more challenging cataract surgery, ECCE and ICCE cases.

In our study, 92.9% of all cataract cases and 97.1% after ocular comorbidity was omitted, achieved post-operative vision better than 6/18. On further analysis, we found that 88.5% of all cataract cases can obtain post-operative VA better than 6/12. After ocular comorbidity was excluded, 94.7% of the cases successfully achieve VA better than 6/12 (Table 1). This result is comparable with other cataract centres in the United States of America, Europe, and Asia countries [10–14]. The United Kingdom National Cataract Survey and Cataract National Dataset Electronic Multicentre Audit revealed 86%–91% cataract patients achieved postoperative vision 6/12 or better and the result is further improved to 92%–95% after excluding ocular comorbidity [11, 12].

The limitation of this study is that it was a retrospective study that limits the information available for analysis. Nevertheless, the number of risk factors related to cataract surgery visual outcome identified in our study was larger than in previous studies. Information bias could have occurred in a retrospective study. However, our study was a nationwide population-based study. All age-related cataract surgeries which were performed in the public hospital under the MOH between 2013 and 2017 were included. The cohort members were representative of the population of all patients with age-related cataract surgeries as the public sector is the largest healthcare provider. Hence, selection bias was minimised.

## Conclusion

A good visual outcome is achieved after cataract surgery in most cases. In our study, elderly patients (80 years and above) with pre-existing vision worse than 6/60, systemic and ocular co-morbidities who underwent combined cataract surgery, ECCE or ICCE with ACIOL implantation under general or regional anaesthesia with intra- or post-operative complications had the poorer visual outcome.

This large multicentre multi-ethnic database study identifies risk factors for cataract surgery visual outcome. It could serve as a benchmark for the standard of care and a basis for an evidence-based guideline among the Malaysian population.

## Supporting information

S1 Table Post-operative BCVA for patients who underwent phacoemulsification / ECCE / ICCE with and without ocular comorbidity. Most of the patients achieved good postoperative vision regardless of the types of surgery (phacoemulsification / ECCE / ICCE). The vision improve further after the ocular comorbidities were excluded.
(DOCX)
