# Supplementary material for: Risk factors affecting cataract surgery outcome: The Malaysian cataract surgery registry
Source: PLoS One. 2022 Sep 21;17(9):e0274939. doi: 10.1371/journal.pone.0274939 (PMC9491522; doi:10.1371/journal.pone.0274939)
Supplement: S1 Table — Most of the patients achieved good postoperative vision regardless of the types of surgery (phacoemulsification / ECCE / ICCE). The vision improve further after the ocular comorbidities were excluded. (DOCX) [file pone.0274939.s001.docx]

**S1 Table. Post-operative BCVA for patients who underwent phacoemulsification / ECCE / ICCE with and without ocular comorbidity**

|  | **Phacoemulsification** | | | | **ECCE** | | | | **ICCE** | | | |
| --- | --- | --- | --- | --- | --- | --- | --- | --- | --- | --- | --- | --- |
| **Outcomes** | **All phaco (n=120,001)** | | **All phaco excluding ocular co-morbidities**  **(n=77,436)** | | **All ECCE**  **(n=10,920)** | | **All ECCE excluding ocular co-morbidities**  **(n=4821)** | | **All ICCE**  **(n=271)** | | **All ICCE excluding ocular co-morbidities**  **(n=74)** | |
|  | **n** | **(%)** | **n** | **(%)** | **n** | **(%)** | **n** | **(%)** | **n** | **(%)** | **n** | **(%)** |
| **Postoperative BCVA** |  |  |  |  |  |  |  |  |  |  |  |  |
| Good (>6/18) | 112,623 | (93.9) | 75,582 | (97.6) | 9,099 | (83.3) | 4,346 | (90.1) | 174 | (64.2) | 58 | (78.4) |
| Borderline (6/18–6/60) | 5,310 | ( 4.4) | 1,506 | ( 1.9) | 1,314 | (12.0) | 397 | ( 8.2) | 61 | (22.5) | 11 | (14.9) |
| Poor (<6/60) | 2,068 | ( 1.7) | 348 | ( 0.5) | 507 | ( 4.6) | 78 | ( 1.6) | 36 | (13.3) | 5 | ( 6.8) |
|  |  |  |  |  |  |  |  |  |  |  |  |  |
| >6/12 | 107,941 | (90.0) | 73,889 | (95.4) | 8,106 | (74.2) | 4,009 | (83.2) | 139 | (51.3) | 50 | (67.6) |
|  |  |  |  |  |  |  |  |  |  |  |  |  |
| 6/6 or better | 52,484 | (43.7) | 37,959 | (49.0) | 2,268 | (20.8) | 1,180 | (24.5) | 31 | (11.4) | 9 | (12.2) |

Most of the patients achieved good postoperative vision regardless of the types of surgery (phacoemulsification / ECCE / ICCE). The vision improve further after the ocular comorbidities were excluded.
